# The Epigenetic Profile of Tumor Endothelial Cells. Effects of Combined Therapy with Antiangiogenic and Epigenetic Drugs on Cancer Progression

**DOI:** 10.3390/ijms21072606

**Published:** 2020-04-09

**Authors:** Oskar Ciesielski, Marta Biesiekierska, Baptiste Panthu, Varvara Vialichka, Luciano Pirola, Aneta Balcerczyk

**Affiliations:** 1 Department of Molecular Biophysics, Faculty of Biology and Environmental Protection, University of Lodz, Pomorska 141/143, 90-236 Lodz, Poland; oskar.ciesielski2@unilodz.eu (O.C.); marta.biesiekierska@unilodz.eu (M.B.); varvara.vialichka@unilodz.eu (V.V.); 2 The Bio-Med-Chem Doctoral School of the University of Lodz and Lodz Institutes of the Polish Academy of Sciences, University of Lodz, Banacha 12/16, 90-237 Lodz, Poland; 3 INSERM Unit 1060, CarMeN Laboratory, Lyon 1 University, 165 Chemin du Grand Revoyet—BP12, F-69495 Pierre Bénite CEDEX, France; baptiste.panthu@univ-lyon1.fr (B.P.); luciano.pirola@univ-lyon1.fr (L.P.)

**Keywords:** tumor endothelial cells, epigenetics, cancer, metastasis, antiangiogenic treatment, epi-drugs

## Abstract

Tumors require a constant supply of nutrients to grow which are provided through tumor blood vessels. To metastasize, tumors need a route to enter circulation, that route is also provided by tumor blood vessels. Thus, angiogenesis is necessary for both tumor progression and metastasis. Angiogenesis is tightly regulated by a balance of angiogenic and antiangiogenic factors. Angiogenic factors of the vascular endothelial growth factor (VEGF) family lead to the activation of endothelial cells, proliferation, and neovascularization. Significant VEGF-A upregulation is commonly observed in cancer cells, also due to hypoxic conditions, and activates endothelial cells (ECs) by paracrine signaling stimulating cell migration and proliferation, resulting in tumor-dependent angiogenesis. Conversely, antiangiogenic factors inhibit angiogenesis by suppressing ECs activation. One of the best-known anti-angiogenic factors is thrombospondin-1 (TSP-1). In pathological angiogenesis, the balance shifts towards the proangiogenic factors and an angiogenic switch that promotes tumor angiogenesis. Here, we review the current literature supporting the notion of the existence of two different endothelial lineages: normal endothelial cells (NECs), representing the physiological form of vascular endothelium, and tumor endothelial cells (TECs), which are strongly promoted by the tumor microenvironment and are biologically different from NECs. The angiogenic switch would be also important for the explanation of the differences between NECs and TECs, as angiogenic factors, cytokines and growth factors secreted into the tumor microenvironment may cause genetic instability. In this review, we focus on the epigenetic differences between the two endothelial lineages, which provide a possible window for pharmacological targeting of TECs.

## 1. Introduction 

According to reports from the World Health Organization, in developed countries, cardiovascular disease and cancer are the two medical conditions with the highest incidence. In both cases, dysfunction of the vascular endothelium, the layer of cells lining the interior of the blood and lymph vessels, is crucial for the progression of these pathological states. Over the years, it was thought that the endothelium is merely a passive barrier between the blood and the surrounding tissues [1]. However, the current state of knowledge shows that the vascular endothelium is a dynamic, “dispersed endocrine organ” spread over an area of approximately 7 m^2^ that controls: (i) the flow of blood, (ii) organism nutrition by the passage of metabolites and gases exchange, (iii) immunity by circulating immune cells and antibodies, as well as (iv) trafficking of leukocytes [2,3,4]. Moreover, endothelial cells are involved in several physiological processes including (v) angiogenesis, (vi) blood coagulation and fibrinolysis, (vii) vasomotor and blood pressure regulation, and (viii) inflammatory reactions, which are conditioned by a multitude of synthesized and released hormonal and chemical mediators, including vascular endothelial growth factor (VEGF), nitric oxide (NO^•^), or prostaglandin E2 [5,6,7].

Dysfunction of the endothelium results in a number of disorders. In tumors, endothelial cells due to chronic stimulation by growth factors and hypoxia, change their phenotype into tumor endothelial cells (TECs), which results in abnormalities in tumor blood vessel morphology. TECs are fragile and leaky as compared to normal vessels, that in consequence alters blood flow. These abnormalities in the tumor endothelium contribute to tumor growth and metastasis [8,9]. Thus, a detailed understanding of TECs biology may open a new window in anticancer therapy and facilitate effective antiangiogenic treatment.

In addition to TECs, cancer-associated fibroblasts (CAFs) are yet another component of the tumor microenvironment that contributes to tumor remodeling and mediate the interaction with tumor-infiltrating immune cells.Moreover, CAFs have been shown to promote tumor angiogenesis by overexpressing WNT2 (Wnt family member 2) in colorectal cancer [10,11,12]. The understanding of CAFs biology and potential targeting strategies directed to CAFs constitute a relatively new and highly dynamic field of investigation. Recent excellent reviews summarize the state-of-the-art in this expanding field that will not be discussed further in this review [13,14].

Here, we provide an overview of the current literature regarding the epigenetic background of TECs biology, as well as recently performed (and ongoing) clinical trials targeting tumors on the bases of combined treatment with antiangiogenic and epigenetic drugs.

### Literature Search

Relevant literature was retrieved from Pubmed (https://www.ncbi.nlm.nih.gov/) and, for the identification of the clinical trials, from www.clinicaltrials.gov. The following keywords, alone or in combination, were used, e.g., tumor endothelial cells, tumor microenvironment, neoangiogenesis, DNA methylation, histone posttranslational modifications, HDACs, cancer-associated fibroblasts. Collectively, these search terms yield thousands of results on Pubmed and were filtered for their relevance to the key aspect covered in this review, i.e., the specificity of TECs versus NECs. In some instances, relevant literature within references retrieved from Pubmed was cited.

## 2. NECs versus TECs

Normal endothelial cells (NECs) are diploid cells forming regular monolayers that are able to create capillary-like tubes. Under physiological conditions, endothelial cells become activated according to environmental needs, such as stopping the hemorrhage, during wound healing or vascular damage repair. NECs are normally quiescent and one of the most genetically stable cells of the body, with a turnover time exceeding hundreds of days. Generally, NECs regulate multiple vascular processes among which vascular permeability, leukocyte trafficking, regulation of vascular tone and immunity can be distinguished. Heterogeneity of NECs is related to both intrinsic (i.e., genetic) factors and extrinsic factors, encompassing cell to cell contact, cell–matrix interactions, mechanical forces (i.e., shear stress), pH, and pO_2_ [10]. NECs engaged in vessels formation can be divided into three subtypes: (i) migratory “tip” cells which lead the sprouting of new vessels, (ii) proliferative “stalk” cells mediating the elongation of the newly formed sprout, and (iii) “phalanx” cells, a quiescent EC lineage lining the interior of the newly-formed vessel [15].

Tumor blood vessels demonstrate an abnormal phenotype and, consequently, important morphological changes. Unlike NECs, TECs are irregular in shape and size with long cytoplasmic projections extending across the vessel lumen [16]. Enlarged vessels with severe branching alongside gaps and fenestrae present in the walls of tumor blood vessels lead to hemorrhage, low tissue perfusion and plasma leakage [17]. Due to these abnormalities, not only the unbalanced expression of angiogenic factors is observed, but also tumor cells intravasation, which is an initial step in metastasis. [18].

Recent studies have demonstrated many differences between NECs and TECs at the molecular level. In terms of responsiveness to growth factors such as adrenomedullin, VEGF (vascular endothelial growth factor), EGF (epidermal growth factor), and FGF (fibroblast growth factor), TECs exhibit several abnormalities contributing to their proangiogenic and antiapoptotic phenotype [19], which is not observed in NECs. Additionally, TECs are characterized by upregulated aldehyde dehydrogenase (ALDH) expression in comparison to NECs. Moreover, it was shown that ALDH^high^ TECs form significantly more tubes on the matrigel basement membrane matrix even under serum starvation in comparison to ALDH^low^ TECs. This suggests a direct relationship between ALDH and the enhanced proangiogenic phenotype of TECs, although the subject still needs further research [20].

TECs express embryonic markers, e.g., the renal transcription factor PAX2 [21], which is a general characteristic of tumor cells [16]. Cytogenetically, TECs show abnormalities in the form of aneuploidy and abnormal centrosomes, Table 1. Even though such features have been observed in mouse tumors [22] and human renal carcinomas [23], respectively, it has been also confirmed that these chromosomal aberrations were not artifacts caused by tumor cell contaminants. Cytogenetic abnormalities indicate genetic instability, which may explain the observed resistance of TECs to chemotherapeutics, i.e., vincristine, 5-fluorouracil, adriamycin, and paclitaxel [24]. However, there are no data explaining the aforementioned resistance mechanism.

### 2.1. The Multiple Origins of TECs

**Figure 1:**
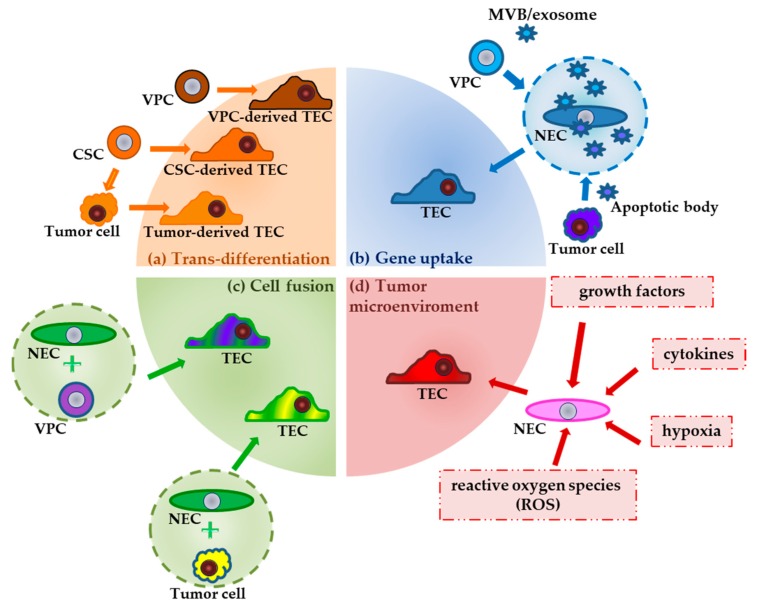
Mechanisms of cytogenetic transformation of NECs to TECs. Proposed mechanisms: (**a**) Trans-differentiation: TECs arise from tumor cells, cancer stem cells or vascular progenitor cells. (**b**) Gene uptake: NECs can accept oncogenes by phagocytosis of apoptotic bodies or exosomes, released by tumor cells or endothelial progenitor cells. (**c**) Cell fusion: VPC or tumor cells can fuse with normal endothelial cells. (**d**) Tumor microenvironment: hypoxia, reactive oxygen species (ROS), growth factors, or cytokines in the tumor microenvironment may be another factor causing genetic instability. CSC—cancer stem cell; MVB—multivesicular body; NEC—normal endothelial cell; TEC—tumor endothelial cell; VPC—vascular progenitor cell.

There are several mechanisms causing cytogenetic abnormalities in TECs. Vascular progenitor cells (VPCs) and tumor cells, previously formed from cancer stem cells, can directly transdifferentiate into specific TECs or fuse with NECs and then transform into TECs (Figure 1). Endothelial progenitor cells (EPCs) or tumor cells can release exosomes and apoptotic bodies to allow ECs to take up human tumor oncogenes by phagocytosis. Hypoxia, reactive oxygen species (ROS), growth factors or cytokines released in the tumor microenvironment may be another factor causing genetic instability [24].

Tumor blood vessels are often fenestrated, immature, and leaky. This leads to high tissue pressure within the tumor, compression of the blood vessels, and, in turn, hypoxia [32,33]. Hypoxia triggers the overproduction of VEGF, excessive vascular permeability, and genetic instability in TECs [34]. Moreover, the morphology and pericyte coverage of tumor blood vessels differ between tumor type and progression stage. Blood vessels of highly metastatic (HM) tumors are more immature and possess fewer pericytes in comparison to blood vessels of low metastatic (LM) tumors. This contributes to the higher hypoxic state of HM than LM tumors [35]. TECs isolated from HM tumors (HM-TECs) exhibit higher proliferative index, motility, resistance to anticancer drugs (5-fluorouracil, paclitaxel), and chromosomal abnormalities in terms of aneuploidy and karyotype in comparison to TECs isolated from LM tumors [24].

As the basic function of centrosomes is to establish cell polarity and properly segregate the chromosomes, TECs possessing abnormal centrosomes (in a number between one and five) result in loss of polarity and disaggregation, usually detected in aggressive human malignant tumors [22].

### 2.2. Metabolic Reprogramming in NECs and TECs

Intrinsic and extrinsic features of the tumor microenvironment influence the metabolic heterogeneity between TECs and NECs. Intrinsic features within a cell include genetic, epigenetic, and metabolic changes, whereas extrinsic features concern nutrient and oxygen availability, waste product removal, and pH gradient determined by the tumor microenvironment [36].

Analysis of the metabolic profile of TECs has been mainly performed for their metabolite capacity to promote cell proliferation or to support the synthesis of the cell’s building blocks such as amino acids or nucleotides. In the available literature, TECs are described as a hyperglycolytic lineage [37,38]. Lactic acid produced by TECs, or released by tumor cells, stimulates TEC proliferation, as demonstrated by increased carbonic anhydrase II (CAII) expression [39].

**Figure 2:**
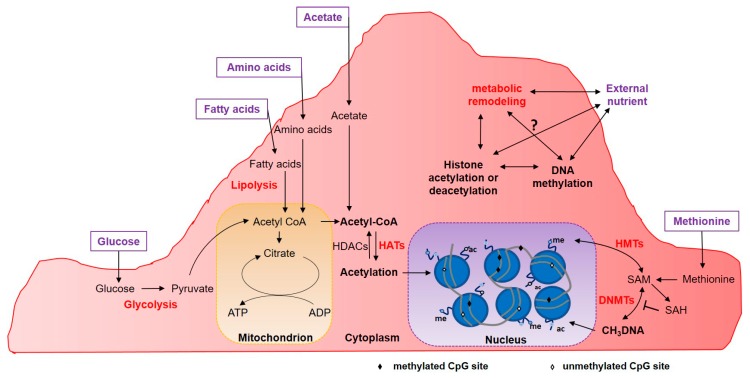
Metabolic reprogramming of EC derived from the intrinsic and extrinsic microenvironment. Acetyl-CoA derived from glucose, fatty acid, amino acid or acetate metabolism is the substrate for histone acetylation driven by HATs. Metabolic remodeling can upregulate enzymatic activity from glycolysis, lipolysis and other pathways leading to overproduction of acetyl-CoA in the cytoplasm. DNA methylation is governed by the availability of dietary methionine, that enters a cycle due to its conversion into SAM, a donor of the methyl groups. DNMTs—DNA N-methyltransferase; HATs—histone Acetyl-Transferase; HDACs—histone deacetylase; HMTs—histone methyltransferases; SAM—*S*-adenosyl-methionine; SAH—*S*-adenosyl-homocysteine.

NECs require specific metabolic adaptations to grow, and during the angiogenic switch to angiogenesis, they undergo metabolic reprogramming [37]. Although NECs are exposed to high concentrations of oxygen in the blood, when sprouting, their oxygen consumption is lower than in any other cell type [40]. Hence, during sprouting, glycolysis is the major metabolic determinant in the specification and functioning of the tip and stalk NECs [41]. Fatty acid (FA) metabolism is also a substantial contributor to NECs proliferation. Fatty acid-binding proteins (FABPs), especially FABP4, are target genes of VEGF and mediate the proliferation and sprouting of NECs via the regulation of the uptake and intracellular trafficking of free fatty acids and cholesterol [42]. Therefore, highlighting an increase of glycolysis coupled to lactogenesis to the detriment of the mitochondrial respiration as a major source of energy defines the proliferating NECs. Glycolysis also provides intermediates for the branched pentose phosphate pathway leading to the anabolic synthesis of nucleotides. Overall, higher glycolysis increases the acetyl-CoA pool that could be used for histone acetylation and concomitant gene expression remodeling (Figure 2). Interestingly, recent studies have shown that in ECs unable to use fatty acids due to CPT1A silencing (an enzyme limiting fatty acid oxidation), acetate supplementation enhances NECs proliferation, by replacing the fatty acids-derived pool of acetyl-CoA [43]. Independently, studies focused on the role of diet-microbiota interactions in epigenetic remodeling have revealed that acetate supplementation feeds cells for acetyl-CoA production, some of which is used as a substrate for histone acetylation in liver and colon [44]. As of today, studies that show a direct role of nutrient supplementation or depletion and the effect on histone modifications upon TECs epigenetic reprogramming are missing. The same question will be worth addressing relative to DNA methylation through the *S*-adenosyl-methionine transmethylation pathways and related external nutrients [45]. It is critical to underscore that the metabolic profiles of TECs differ from those of NECs, with the glycolytic process being particularly upregulated in TECs. TECs are rapidly proliferating ECs and such proliferation is supported by increased glucose uptake. It was observed that even partial inhibition of glycolysis induces tumor vessel normalization, suggesting that modulation of TEC metabolism through targeting glycolysis may be a promising therapeutic strategy [36]. From a mechanistic point of view, the direct or indirect relationship between the external environment and especially nutrient availability with the epigenetic changes during TECs progression remains an open field of investigation.

## 3. Epigenetic Profiling of Tumor Endothelial Cells

Epigenetic mechanisms play a key role in determining the gene expression profile in cells, mainly by affecting chromatin accessibility to transcription factors [46,47]. Covalent epigenetic modifications, i.e., DNA methylation on cytosines within the CpG dinucleotide sequence and histone posttranslational modifications (PTMs) provide a combinatorial platform to determine the chromatin compaction state. Another epigenetic mechanism of gene regulation, acting in concert to the DNA/histone covalent modifications, is the micro-RNA associated control of gene expression, which mainly relays a silencing response. Aberrant epigenetic regulation is frequently observed in cancer cells, where gene silencing of tumor suppressors is driven by DNA methylation and histone deacetylation of promoter regions, and overexpression of oncogenes depends on DNA hypomethylation [48,49]. The knowledge of the epigenetic profile of cancer cells is now rather well established. The most common epigenetic changes in cancer include promoter-specific DNA hypermethylation and gene silencing, as the downregulation of the *EN1* homeobox gene observed in various cancers [50,51,52]. Epigenetic modifications were shown to occur in key oncogenes as well as suppressor genes, providing evidence that they are essential in driving tumorigenesis [53]. Epigenetic changes are also essential in the functioning of normal ECs as proven by numerous works [54,55,56]. For instance, the expression of the flagship enzyme synthesized by ECs—eNOS (endothelial nitric oxide synthase) is governed by a specific DNA methylation pattern [57]. A growing body of evidence suggests that the main functions of NECs are tightly regulated by epigenetics and that aberrant changes disrupting normal gene expression may lead to pathological states, such as type 2 diabetes [58] and atherosclerosis [59]. In contrast, the aberrant epigenetic modulation of TECs is only partly understood. Here, we summarize the available literature on the epigenetic profile of TECs, we address the epigenetic phenomena that contribute to the NEC/TEC switch, and we discuss the underpinnings of these modifications on the tumor–endothelium relationship.

### 3.1. The DNA Methylation Profile of TECs

DNA methylation influences gene expression by affecting the DNA-chromatin and DNA-transcription factors interactions [60]. While, in somatic cells, methylation patterns are very stable, cancer cells exhibit aberrant DNA methylation profiles. In NECs, DNA methylation is an important contributor to endothelial-specific transcription patterns. The *eNOS* promoter region is hypomethylated in NECs as compared to vascular smooth muscle cells and accounts for the endothelial-specific expression of *eNOS*. Indeed, pharmacological inhibition of DNA methyltransferases (DNMTs) in non-endothelial cell types resulted in increased *eNOS* expression [61]. DNA hypomethylation was identified on most endothelial genes such as *VWF* (von Willebrand factor), *CD31*, *CDH5* (VE-cadherin). However, the VEGF receptors *VEGFR-1* and *VEGFR-2* displayed no hypomethylation [62]. On a larger scale microarray analysis, Hellbrekers et al. analyzed down-regulated genes in tumor conditioned versus quiescent ECs and assessed the ECs response to treatment with 5-aza-2′-deoxycytidine (DAC) and Trichostatin A (TSA) [63]. Tumor-conditioned media-induced gene expression alteration of 81 genes, 77% of which contained a CpG island in the transcription site or near the upstream region. Treatment of tumor-conditioned ECs with DAC and TSA restored the expression of selected genes, which suggests that their expression may be modulated via DNA methylation and histone acetylation. This study identified a set of angiogenesis-suppressing genes that become silenced upon the treatment of ECs with tumor-conditioned media. Mechanistically, the silencing occurred in conjunction with promoter hypermethylation and histone deacetylation, and gene reactivation by DNMT and HDAC inhibitors led to the reversal of these epigenetic modifications, suggesting a mechanism for epigenetic regulation of tumor angiogenesis by tumor-secreted factors [63].

Critically, silencing of some genes involved in the regulation of endothelial cells functioning like growth and sprouting: clusterin (*CLU*), fibrillin (*FBN1*), tetraspanin 2 (*TSPAN2*), and tumor necrosis factor receptor superfamily member 21 (*TNFRSF21*), is mainly the effect of histone modifications and not of changes in the DNA methylation profile, as no methylated CpG regions were identified neither in control ECs nor in conditioned media-treated ECs [63]. Therefore, gene silencing of this gene subset may not be due to increased promoter methylation. Yet, the re-induction of gene expression by DAC suggests that transcriptional control by DNA methylation might also occur in regulatory regions located outside of promoters. For example, hypermethylation of the clusterin gene was reported within a CpG island located 14.5 kb upstream of the gene. Another study by the same group showed that in tumor-conditioned HUVECs (TC-HUVECs), the expression of Intracellular Adhesion Molecule 1 (ICAM-1) protein was down-regulated by 81% when compared to normal HUVECs, and decreased expression of Vascular Cell Adhesion Molecule 1 (VCAM-1) and E-selectin was also reported. Treatment of TC-HUVECs with DNMTs inhibitors DAC and zebularine significantly increased ICAM-1, VCAM-1, and E-selectin expression. ICAM-1 is the primary endothelial adhesion molecule and its regulation is important to ECs anergy, the induction of its re-expression via DAC, zebularine, and TSA inhibitors in TC-HUVECs resulted in restored leukocyte adhesion [64]. Widespread differences in methylation and transcriptional patterns were observed between TECs derived from malignant human prostate cancer and NECs [65]. 5490 differentially methylated loci were identified in TECs versus NECs comparison, including 3 genes, *JMY*, *EPB41*, and *GMNN*, whose differential methylation between the two ECs types was further confirmed by bisulfite sequencing. The study confirmed that the DNA methylation profile of TECs drastically differs from that of NECs, as 548 and 179 differentially expressed genes due to DNA methylation were observed, for African-American and Caucasian-American groups, respectively [65]. Differential gene promoter methylation of 25-hydroxyvitamin D-24-hydroxylase/*CYP24A1* was also observed between benign and malignant human prostate endothelium [66]. The aforementioned findings are consistent with previous observations obtained also from other tumor types [67,68]. The important role of DNA methylation in regulating TECs-driven tumor metastasis was recently investigated by utilizing high metastatic tumor endothelial cells (HM-TECs) [69].

TECs acquired specific features due to their surrounding environment, including the demethylation of the promoter of biglycan, a molecule that is an important component of the extracellular matrix and it was also found that its high level promotes angiogenesis and colon tumor growth [70]. HM-TECs co-implanted with low metastatic lung tumor cells accelerated tumor metastasis. The observed accelerated metastasis was the direct result of demethylation of the biglycan promoter region, its upregulated expression and activated tumor cell migration via biglycan-dependent activation of NF-κB and extracellular signal-regulated kinase 1/2 [71].

### 3.2. Histone Posttranslational Modifications

The combinatorial occurrence of the many possible histone PTMs on the chromatin template generates the so-called histone code, that is read by proteins modulating chromatin structure and, consequently, gene expression [72]. In concert with DNA methylation, histone PTMs modulate gene expression. In cancer cells, histone PTMs contribute to the silencing of suppressor genes, although DNA methylation events seem to dominate over histone PTMs in maintaining the silent state of suppressor genes, as multiple studies show that silenced genes can be reactivated by DAC alone. Histone tail PTMs can be distinguished into two groups, respectively associated with transcriptionally active or repressed chromatin. Hyperacetylation of lysine residues on the core histones H3 and H4 are generally considered as an active chromatin mark, whereas deacetylation is a mark of inactive genes [73,74]. The transcriptional effects of histone methylation depend on the location of modified residue and the number of methyl groups added; histone H3K4 mono-, di-, and tri-methylation are considered as an active mark and H3K9 mono-, di-, and tri-methylation the opposite [75,76]. The roles of the HATs and HDACs in regulating the histone acetylation balance in NECs are well established. It was shown that shear stress enhances HATs activity in ECs, and shears stress-induced expression of eNOS is highly dependent upon increased HAT p300 activity [77]. HADAC3 and 5 were both shown to act as angiogenesis repressors as well as modifiers of the angiogenic gene expression pattern of ECs [78,79]. Moreover, it was shown that in NECs, HDACs modulate the functioning of important cellular pathways, with HDAC1 and HDAC2 controlling TGF-β signaling during endothelial to hematopoietic transition [80] and HDAC7 controlling ECs cell growth through the β-catenin pathway [81]. Several reports support the notion that histone methylation also plays an essential role in regulating the function of ECs. A specific combinatorial histone code that includes the tri-methylation of H3K9, di-methylation of H3K4, as well as acetylation of H3K9 and H4K12, governs the expression of eNOS in NECs [57]. Several histone methyltransferases were shown to impact the physiological functioning of the endothelium. Suppression of the EZH2 methyltransferase via administration of excess of *S*-adenosyl-L-homocysteine (SAH) contributed to NF-κB activation and subsequent vascular inflammatory response [82]. Global pharmacological inhibition of arginine and lysine methyltransferases via AMI-1 and AMI-5 compounds induced an angiostatic profile in HMEC-1 and HUVECs. Histone methyltransferase (HMT) inhibition directly affected the proliferation of ECs by suppressing cell cycle progression in the G_0_/G_1_ phase [83]. Cell cycle arrest in the G_1_ phase was also observed by inhibiting the HMT G9a, by either BIX-01294 or chaetocin pharmacological inhibitors, and shRNA knockdown [84]. Histone methylation was also shown to be an important mark of angiogenesis, as knockdown of HMT DOT1L (a histone H3K79 methyltransferase) in HUVECs resulted in decreased cell viability, tube and capillary sprout formation [85]. The ability to form functional vascular networks by NECs in vivo was also reduced, as it was mediated by H3K79me2 cooperating with the ETS-1 transcription factor to regulate VEGFR2 expression [85].

Collectively, these studies suggest that histone modifications play a key role in maintaining the function of normal endothelial cells, however, the knowledge of the profile of these modifications in the tumor endothelium remains relatively limited. Nonetheless, the study by Hellebrekers et al. showed that histone modifications of the promoter regions play a role in silencing genes in TECs, with no apparent role for DNA methylation [63]. As discussed above, promoter acetyl-histone H3 levels were significantly decreased for several genes involved in the regulation of endothelial cell biology, as *CLU* (clusterin), *FBN1* (fibrillin 1), *TSPAN2* (tetraspanin 2), *TNFRSF21* (tumor necrosis factor receptor superfamily member 21), *QSCN6* (quiescin Q6), *ICAM-1* (intercellular adhesion molecule 1), and *IGFBP3*, in TC-HUVECs compared to normal HUVECs arguing these genes as negative regulators of endothelial cell growth and angiogenesis. Changes in the methylation status of H3K4 were also observed, with a significant decrease in methylation reported in TC-HUVECs [63]. Summarizing, gene silencing in TC-HUVECs was tightly regulated by histone modifications, selected genes were repressed by promoter histone H3 deacetylation and loss of H3K4 methylation, but not by DNA methylation [63]. The anergy of tumor endothelial cells and the loss of leukocyte-endothelial cell adhesion in vitro is the direct result of ICAM-1 down-regulation, the silencing is also regulated by the aforementioned modulation of the histone modification profile [64]. The ability of DNMT and HDAC inhibitors to restore leukocyte-endothelial cell adhesion was also observed on an in vivo tumor vessel model. Leukocyte–vessel interactions were quantified by intravital microscopy on immunocompetent B16F10 melanoma-bearing C57BL/6 mice model. In mice treated with TSA, leukocyte adhesion, and leukocyte rolling were significantly increased when compared to untreated tumors [64]. These results provide an important insight into how epigenetic regulation of TECs modulates their functioning, and how HDAC inhibitors may be utilized in therapy for the reversal of endothelial cell anergy.

### 3.3. miRNAs in Regulating NECs/TECs Metabolism

Non-coding microRNAs (miRNAs) are 20–24 nucleotides-long RNA molecules that act mainly as negative regulators of post-transcriptional gene expression. miRNAs exert their transcriptional modulatory function by binding to cognate sequences—not necessarily fully complementary—on messenger RNAs. miRNA-mediated gene silencing is important for regulation of endothelial function, and dysregulation of miRNA-dependent transcriptional control can lead to metabolic dysfunctions and disruption of ECs homeostasis in metabolic disease [86] and cancer [87]. miRNA-126 is an important endothelial-specific miRNA involved in maintaining vascular integrity and angiogenesis of NECs [88,89]. miRNA-126 regulates the VEGF pathway by targeting sprouty-related protein 1 [89] and phosphoinositide-3 kinase [89,90]. The involvement of miRNA-126 in angiogenic pathways was also demonstrated by a study in which a miRNA-126 specific inhibitor (antagomir-126) was shown to impair ischemia-induced angiogenesis [91]. Other key miRNAs involved in the regulation of endothelial functions include miRNA-155 which targets eNOS and regulating the vascular tone [92], and miRNA-125b targeting VE-cadherin-5 and therefore inhibiting the formation of vessel tubes [93]. Many other miRNAs were identified as contributors to endothelial cells physiopathology, as covered by recent reviews [94,95] and reviewed in Table 2.

In recent years, an increasing number of studies have shown that miRNAs are important mediators of the interaction between tumor and endothelial cells [96]. In cancer cells, the expression of miRNAs is considerably altered compared to normal cells and also often influenced by stromal cells [87]. This influence is reciprocal as the expression of miRNAs in stromal cells and ECs is modulated by tumor cells. This modulation of miRNA expression in ECs by tumor cells was observed in ECs co-cultured with hepatocarcinoma cells, mainly due to the secretion of soluble growth factors and cytokines [97,98]. Once overexpressed in cancer cells, part of the miRNA pool can be shuttled to TECs and also vice versa by the means of macrovesicles, exosomes, or gap junctions [99,100]. These miRNAs are involved in regulating angiogenesis, intravasation, extravasation, and tumor progression. miRNA-210 was observed to enhance angiogenesis when transferred from breast cancer cells to TECs via neutral sphingomyelinase 2 (nSMase2)-dependent exosome transfer [101]. miRNA-25-3p, another pro-angiogenic miRNA transferred to NECs via exosomes targets VEGFR2, occludin and claudin-5 expression [100]. Anti-angiogenic miRNAs also exert their function when transferred to TECs. The overexpression of miRNA-29c in glioma cells resulted in reduced VEGF expression and subsequent secretion, HUVECs cultured in the tumor conditioned medium display a decreased number of tubular structures [102]. Similarly, exosomes derived from mesenchymal stem cells (MSCs) and containing miR-16, a miRNA targeting VEGF, contribute to the anti-angiogenic effect of MSC-derived exosomes. Administration of MSC-derived exosomes lowered VEGF secretion by the mouse breast cancer cell line 4T1 in a dose-dependent manner [103]. Several other miRNAs that play a significant role in the communication between cancer cells and TECs are summarized in Table 2.

## 4. Role of TECs in Promoting the Metastatic Phenotype of Cancer Cells

Over time, tumor cells gain malignant potential and begin to initially invade the surrounding stromal cells and extracellular matrix. At a later stage, to reach distant organs, tumor cells intravasate into blood vessels. The role of TECs is not only limited to supplying oxygen and nutrients to the growing tumor, but they are also essential for tumor progression and metastasis. TECs exhibit upregulated expression of VEGF receptors, which contributes to their rapid response and facilitates the formation of disorganized blood vessels, which serve as a gateway for tumor cells to reach the bloodstream [131]. After stimulation with VEGF, VE-cadherin is internalized and the tight junctions between ECs loosen, inducing vascular permeability [132]. Moreover, immature and disorganized tumor blood vessels lack smooth muscle cells and pericytes, which makes it easier for tumor cells to permeate them through [27].

TECs not only provide a passive entry route for tumor cells but may also actively promote metastasis through the production of “angiocrine factors” [133]. These factors, secreted by TECs into the tumor microenvironment stimulate tumor growth and cell migration. In prostate cancer, TECs secrete IL-6, IL-8, TGFβ, and various other molecules that mediate cancer progression in a paracrine fashion [134]. TEC-derived angiocrine factors were shown to drive tumorigenesis, promote tumor growth, regulate angiogenesis, and stimulate cell intravasation. Concomitantly, Slit2, a tumor-suppressive angiocrine factor was shown to be downregulated in TECs [135]. It was also reported that TECs from high metastatic tumors secrete higher levels of angiocrine factors when compared to TECs from non-metastatic tumors [35], further proving the heterogeneity of TECs. Maishi et al. reported that TECs from high metastatic tumors adhere to and attract cancer cells to a greater extent when compared to TECs from non-metastatic tumors or NECs. The main angiocrine factor responsible for these phenomena in high metastatic TECs was biglycan, which facilitated the migration of toll-like receptor-expressing cells by activating the ERK and NF-κB signaling [71]. As mentioned above, the dysregulation of biglycan expression was the effect of epigenetic alterations. In vivo, biglycan plasma levels were significantly higher than those observed in healthy individuals [71]. TECs also stimulate tumor cell intravasation and metastasis. Notch1 in TECs was shown to promote lung metastasis. Moreover, the elevated expression of Notch1 in TECs is correlated with poor prognosis for melanoma, lung adenocarcinoma, and breast carcinoma, as Notch1 activity facilitates metastasis [136]. CXCR7 regulates CXCL12–CXCR4 mediated tumor trans-endothelial migration, and high expression of CXCR7 in tumor vasculature is linked with tumor progression angiogenesis and metastasis [137,138,139]. Interestingly it was observed that activated TECs expressing high levels of adhesion molecules protect tumor cells from anoikis in the circulation, by binding to and protecting them until they reach distant organs [140]. ECs also modulate the immune response to tumors by acting as antigen-presenting cells (APCs). ECs from the bone marrow of patients with multiple myeloma were found to work as mature APC, being able to do antigen presentation to CD8^+^ T cells and sustaining a Foxp3 + CD8^+^ T cell population expressing anti-inflammatory IL-10 and TGF-β and endowed with pro-tumor activity [141]. This study demonstrates that ECs not only contribute to tumor progression in terms of neovascularization but also by negatively modulating the immune response to the tumor. Taken together, all these findings strongly support the active role of TECs in promoting tumor metastasis.

### TECs and Drug Resistance in Tumors

It is a widely observed phenomenon that, during anti-cancer therapies, administered drugs used become ineffective. This is caused by tumor cells acquiring resistance, by mutation [142], lineage plasticity [143], or due to increased expression of ATP-binding cassette transporters (ABC transporters) favoring the detoxification processes in tumors [144]. Interestingly, it was observed that TECs also display features of drug resistance, which may be conferred to them by surrounding tumor cells. In TECs, the expression level of multidrug resistance protein 1 gene (*MDR1*) and its product P-glycoprotein are highly elevated compared to NECs [145]. In vivo, both human and mouse tumor blood vessels express high levels of P-glycoprotein [145]. Treatment of human microvascular NECs with tumor conditioned medium increased the mRNA levels of *MDR1* [146]. Acquiring drug resistance in TECs is the effect of Akt activation by VEGF secreted from tumor cells as the VEGFR tyrosine kinase inhibitor Ki8751 blocks the upregulation of *MDR1* [145]. Moreover, the tumor microenvironment plays a decisive role in determining the drug resistance of TECs. TECs derived from renal cell carcinoma were shown to be resistant to vincristine treatment [147]. The development of the resistance to adriamycin and 5-fluorouracil was reported in hepatocellular carcinoma-derived TECs [146,148]. TECs may also accelerate the conversion of tumor cells to chemo resistant stem-like tumor cells. Chemotherapy suppresses IGFBP17 in TECs, which leads to the upregulation of IGF1 in tumor cells and activation of the stem cell-like properties of tumor cells [149]. The process of acquiring drug resistance in TECs is an important factor for tumor survival. After angiogenic therapy, the residual resistant TECs may restore angiogenesis and continue to provide nourishment and oxygen to the tumor, in promoting its growth and survival even after chemotherapy.

## 5. Combination of Anti-Angiogenic and Epigenetic Drugs-Based Therapy in Anticancer Treatment: Drug Candidates and Ongoing Clinical Trials

As summarized in our review, recent years brought significant progress into the understanding of the role of epigenetic mechanisms controlling chromatin remodeling and their impact on angiogenesis, neoangiogenesis, and tumorigenesis. The reversible nature of epigenetic modifications makes them a strategic tool in the treatment of many disorders and pathological states. Several epigenetic drugs targeting the activity of DNA methyltransferases as 5-azacytidine or 5-aza-2′-deoxycytidine and histone deacetylases (Suberoylanilide hydroxamic acid, Panobinostat, Belinostat, Romidepsin) have been approved by FDA (the U.S. Food and Drug Administration) for anticancer therapy and many others, especially affecting the histone methylation status, are at the clinical testing stage, including inhibitors of DOT1L methyltransferase (Pinometostat), EZH2 methyltransferase (Tazemtostat, GSK-126 or 3-Deazaneplanocin A), JMJD3 demethylase (GSK-J1, GSK-J4), and LSD1 (GSK-2879552, INCB059872 or INCB057643) [48,150,151].

Although the epi-drugs give promising results in the strategy to curb tumorigenic growth, significantly affecting cancer cell proliferation, and to a certain level the process of new vessels formation (reviewed in [48]), limitations in single epi-drug-based therapy and the need of more efficient neoangiogenesis control, intensified the search for new medicines. This paved the way to multiple clinical trials targeting both tumor cell growth and vasculature in combined epi-drug/angiostatic treatment protocols (an updated summary of these trials is provided in Table 3).

Most of the trials are at the early clinical testing, phase I or II, and the available results of the completed ones do not yet give a clear cut message on the success of the proposed treatments as the success of the proposed therapy depend on multiple factors: type of cancer, stage of the disease, age of patients, additional disorders non-cancer related, duration of exposure to the combination of drugs, doses of drugs given to the patients, etc.

Azacitidine in combination with nivolumab (NCT02397720, phase II) gave promising results in patients with Relapsed/Refractory Acute Myeloid Leukemia (R/R AML) regarding overall survival rate, particularly among salvage 1 and hypomethylation agent-naïve patients. Moreover, the obtained results suggested also that bone marrow immune biomarkers: CD3^+^ and CD8^+^ criteria should be incorporated for patient selection in future trials to increase the success rate of the therapy [152]. In another phase II trial, NCT02546986, examining the efficiency of pembrolizumab + azacitidine (CC-486) treatment, in patients with advanced non-small cell lung cancer (aNSCLC), despite promising preclinical and early clinical studies suggested that the proposed medicines mix can increase survival rate in aNSCLC patients previously subjected to platinum-based therapy, no significant improvement in progression-free survival (primary end-point) was observed with pembrolizumab + azacitidine versus pembrolizumab + placebo [153]. Moreover, no new safety signals were detected for either pembrolizumab or azacitidine, however, gastrointestinal adverse events were more common in the study arm subjected to pembrolizumab + azacitidine treatment [153]. Some of the trials failed due to inaccurate exclusion criteria or high patient heterogeneity, as in the case of the combined therapy based on bevacizumab + everolimus + panobinostat (NCT01055795, phase I), given to the patients with advanced solid tumors at the lowest proposed doses. The trial finished without a positive recommendation for further evaluation as the tested medicines, despite preliminary dose-escalation studies, did not give an acceptable safety outcome. Monitoring of HDACs activity on peripheral mononuclear cells (PMBCs) that was performed during the trial, showed that the applied dose of panobinostat in most of the patients, instead of inhibition, significantly enhanced the activity of deacetylases [154].

Pharmacological studies are also being performed in relation to prolyl hydroxylase domain (PHDs) inhibitors, responsible for the activation of hypoxia-inducible factors (HIFs), the family of transcriptional factors responding to subphysiological concentrations of oxygen [155,156,157]. HIFs activation results in the stimulation of the expression of hypoxia-sensitive genes that are significantly involved in the regulation of cellular metabolism from the level of proliferation or apoptosis [158]. As among HIFs’ target genes are erythropoietin, VEGF, and nitric oxide synthase (NOS), the HIF hydroxylases are an interesting target of anticancer therapy [156,157,159]. Currently, we are aware of four PHDs inhibitors in clinical use, mostly in phase II and III: Daprodustat (GSK1278863), Vadadustat, Molidustat, and Roxadustat (FG4592), however, they are tested for anemia (NCT03029247, NCT03140722, NCT03418168) and Roxadustat for chemotherapy-induced anemia (NCT04076943) [155]. In the early phase of clinical tests, cancer-focused is RO7070179 (EZN-2968), an antisense oligonucleotide targeting HIF-1α (NCT02564614, hepatocellular carcinoma, phase I).

Taken together, the trials investigating the effectiveness of the inhibitors of epigenetic enzymes involved in modulation of DNA methylation and histone posttranslational modifications pattern in combination with angiostatic drugs show the significant potential that remains to be further elucidated. The antiangiogenic drugs used in anticancer combination therapy target key drivers of the angiogenesis process as VEGF, mTOR, or EGFR (Table 3). However, bigger attention is given to the TEC markers and their successful application in the clinic might enhance the success rate of the combined anticancer therapy based on epi-drugs and angiogenesis inhibitors.

## 6. Conclusions and Future Directions

**Figure 3:**
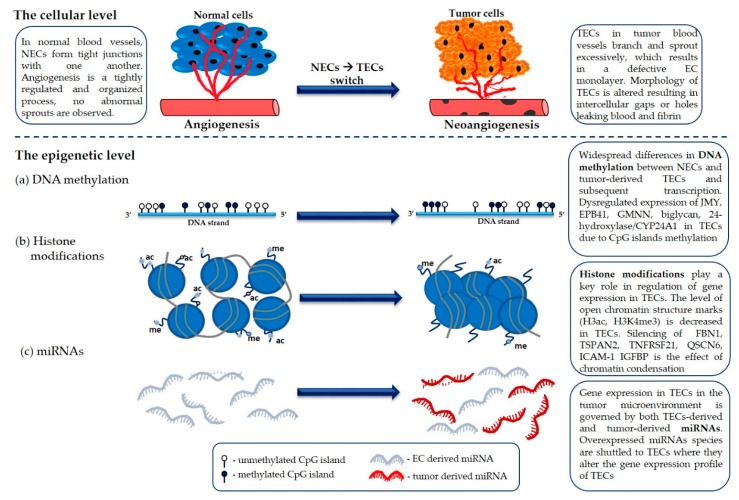
Summary of epigenetic mechanisms including (**a**) DNA methylation, (**b**) histone posttranslational modifications, and (**c**) microRNA, involved in capillary network formation by normal and tumor endothelial cells.

Here, we reviewed the cellular and molecular differences between NECs and TECs that originate from the respective microenvironments including acellular exchange by nutrient or miRNAs and paracrine communication. As discussed in this review, we established that the differences between the two ECs pools heavily rely on epigenetic alterations, as summarized in Figure 3. The fact that TECs are functionally different from NECs provides a therapeutic opportunity to target the tumor vasculature and clinical trials targeting simultaneously tumor cells, as well as the tumor vasculature, are underway. While still in its infancy, the field of investigation on TECs, and on the epigenetic determinant leading to the specificity of TECs versus NECs, provides a promising avenue to target vascularized tumors.

## Figures and Tables

**Table 1:** Summary of the main differences between tumor endothelial cells (TECs) and normal endothelial cells (NECs).

	ECs	TECs	NECs	References
Feature				
**Phenotype**		Proangiogenic, antiapoptotic	Anti-inflammatory, anticoagulative, antifibrotic	[19,20,25,26]
**Morphology**		Irregular	Regular	[27,28]
**Monolayer structure**		Defective with disorganized, overlapping, branches and loosely attached cells	Uniform	[28]
**Ploidy**		Aneuploid	Diploid	[22,23]
**Proliferation time**		Fast	Slow	[29,30]
**Lifespan**		Short	Long	[30]
**Responsiveness to angiogenic growth factors**		High	Low	[20,31]

**Table 2:** miRNAs regulating cancer—TECs cross-talk.

miRNA Released by Cancer Cells	Effect on Angiogenesis Progress	Endothelial Target	Reference
miR-9	Pro-angiogenic	*SOCS5, JAK-STAT* pathway, *COL18A1, THBS2, PTCH1, PHD3*	[104,105]
miR-494	Pro-angiogenic	*PTEN*	[96]
miR-146a	Pro-angiogenic	*PDGFR, VEGF*	[97]
miR-296	Pro-angiogenic	*HGS*	[98]
miR-130a	Pro-angiogenic	*c-MYB*	[106]
miR-93	Pro-angiogenic	*ITGB8, EPLIN, LATS2*	[107,108]
miR-155	Pro-angiogenic	*VHL*	[109]
miR-132	Pro-angiogenic	*P120RasGAP*	[110]
miR-204	Anti-angiogenic	*VEGF, PDGF, JAK2, HIF-1A*	[111]
miR-134	Anti-angiogenic	*VEGF-A, VEGFR1*	[112]
miR-543	Anti-angiogenic	*ANGPT2*	[113]
miR-107	Anti-angiogenic	*HIF-1B, VEGF-A*	[114]
miR-497	Anti-angiogenic	*VEGF-A, VEGFR*	[115,116]
miR-29b	Anti-angiogenic	*MMP2, VEGF-A, PDGF, MMP9, ITGA6, ITGB1, TGFB*	[117,118]
miR-200	Anti-angiogenic	*IL6, CSCL1*	[119]
miR-214	Anti-angiogenic	*HDGF*	[120]
miR-126	Anti-angiogenic	*LRP6, PIK3R2, VEGF-A*	[121,122]
miR-622	Anti-angiogenic	*VEGFA*	[123]
miR-17-92	Pro-angiogenic	*TSP1, DKK3*	[124,125]
	Anti-angiogenic	*EDNRB*	[126]
miR-21	Pro-angiogenic	*VEGF, VEGFR, HIF-1A*	[127]
	Anti-angiogenic	*RhoB*	[128]
miR-27B	Pro-angiogenic	*DLL4/NOTCH*	[129]
	Anti-angiogenic	*VEGF-C*	[130]

Selected miRNAs involved in promoting or repressing angiogenesis (or both) are listed along with their direct or indirect targets. ANGPT2—Angiopoietin 2; c-MYB—MYB transcription factor; COL18A1—Collagen Type XVIII Alpha 1 Chain; CSCL1—C-X-C Motif Chemokine Ligand 1; DKK3—Dickkopf WNT Signaling Pathway Inhibitor 3; DLL4—Delta Like Canonical Notch Ligand 4; EDNRB—Endothelin Receptor Type B; EPLIN—Epithelial Protein Lost In Neoplasm Beta; HDGF—Heparin Binding Growth Factor; HGS—hepatocyte growth factor-regulated tyrosine kinase substrate; HIF1A—Hypoxia Inducible Factor 1 Subunit Alpha; HIF1B—Hypoxia Inducible Factor 1 Subunit Beta; IL6—Interleukin 6; ITGA6—Integrin Subunit Alpha 6; ITGB1—Integrin Subunit Beta 1; ITGB8—Integrin Subunit Beta 8; LATS2—Large Tumor Suppressor Kinase 2; LRP6—LDL Receptor Related Protein 6; MMP2—Matrix Metalloproteinase-2; MMP9—Matrix Metalloproteinase-9; P120RasGAP—P120-Ras GTPase activating protein; PDGF—Platelet-Derived Growth Factor; PDGFR—Platelet Derived Growth Factor Receptor; PHD3—Prolyl-4-hydroxylase domain 3; PIK3R2—Phosphoinositide-3-Kinase Regulatory Subunit 2; PTCH1—Patched 1; PTEN—phosphatase and tensin homolog; RhoB—Ras Homolog Family Member B; SOCS5—Suppressor Of Cytokine Signaling 5; TGFB—Transforming Growth Factor Beta 1; THBS2—Thrombospondin 2; TSP1—Thrombospondin 1; VEGF—Vascular endothelial growth factor; VEGFC—Vascular endothelial growth factor C; VEGFR1—Vascular endothelial growth factor receptor 1; VHL—von Hippel–Lindau Tumor Suppressor.

**Table 3:** Targeting tumor by combined therapy based on epigenetic and angiostatic drugs.

Epi-Drug		Epi-Target		Cancer Type	Clinical Trial Registration Number	Phase of the Study
	Angio-Drug		Angio-Target			
5-Azacitidine		DNTM1		Renal cell carcinoma	NCT00934440	II
Bevacizumab		VEGF				
5-Azacitidine		DNTM1		Metastatic colorectal cancer;	NCT02260440	II
Pembrolizumab		VEGF				
				Advanced solid tumors;	NCT02959437	I/II
				Melanoma and other malignant neoplasms of skin metastatic melanoma;	NCT02816021	II
				carcinoma, non-small lung cancer	NCT02546986	II
5-Azacitidine		DNTM1		AML childhood	NCT03825367	I/II
Nivolumab		VEGF		Acute myeloid leukemia	NCT02397720	II
Decitabine		DNTM1		Childhood solid tumor, childhood lymphoma, relapsed cancer refractory, cancer adult solid tumor, adult lymphoma;	NCT03445858	Early phase I
Pembrolizumab		VEGF				
				relapsed acute myeloid leukemia;	NCT02996474	I/II
				acute myeloid leukemia, high-risk myelodysplastic syndrome;	NCT03969446	I
				Carcinoma, non-small-cell lung cancer	NCT03233724	I/II
Decitabine		DNTM1		Lung cancer, non-small cell lung cancer	NCT02664181	II
Nivolumab		VEGF				
Decitabine, 5-Azacitidine		DNTM1		Acute myeloid leukemia, myelodysplastic syndrome, myelodysplastic syndrome with excess blasts-2	NCT03092674	II, phase III-suspended
Nivolumab		VEGF				
Abexinostat		HDACs: I, II, IV		Metastatic solid tumors	NCT01543763	I
Pazopanib		VEGF				
Vorinostat		HDACs: I, II, IV		Leukemia/myelodysplastic syndromes;	NCT00278330	I
Alvocidib		CDK1, CDK2, CDK4				
				Adult solid tumors	NCT01645514	I
Vorinostat		HDACs: I, II, IV		Lung cancer	NCT02151721	I
Gefitinib		EGFR				
Vorinostat		HDACs: I, II, IV		Advanced cancers	NCT01087554	I
Sirolimus		mTOR				
Panobinostat		HDACs: I, II, IV		Breast cancer	NCT00567879	I
Terastuzumab		Heregulinβ1				
Panobinostat		HDACs: I, II, IV		Advanced solid tumors	NCT01055795	I
Bevacizumab, Everolimus		VEGF mTOR				
Vorinostat		HDACs: I, II, IV		Multiple myeloma;	NCT00858234	I
				Leukemia, Myelodysplastic syndromes;	NCT00818649	II
Bortezomib		VEGF, IL-6, Ang1/2				
				Lymphoma	NCT00810576	II
Tazemetostat		EZH2 HMT		Metastatic bladder urothelial carcinoma, metastatic urothelial carcinoma	NCT03854474	I/II
Pembrolizumab		PD-1				
Tazemetostat		EZH2 HMT		Follicular lymphoma	NCT02220842	I
Atezolizumab (MPDL3280A)		PD-L1, VEGF, Semaphorin4D				
INCB059872		LSD1 KDM		Solid Tumors and Hematologic Malignancy	NCT02712905	I/II
Nivolumab Pembrolizumab		VEGF VEGF				

The clinical trial registration number from www.clinicaltrials.gov is provided. Ang1/2—angiotensin 1/2; CDK1, 2, 4—Cyclin-Dependent Kinase 1, 2, 4; EGFR—epidermal growth factor receptor; mTOR—mammalian target rapamycin; IL-6—interleukin 6; PD-1—programmed death receptor 1; PD-L1—programmed death-ligand 1.
